# The complete mitochondrial genome of an Asian longicorn beetle *Glenea cantor* (Coleoptera: Cerambycidae: Lamiinae)

**DOI:** 10.1080/23802359.2019.1661295

**Published:** 2019-09-06

**Authors:** Xiaoyun Wang, Xialin Zheng, Wen Lu

**Affiliations:** Guangxi Key Laboratory of Agric-Environment and Agric-Products Safety, National Demonstration Center for Experimental Plant Science Education, College of Agriculture, Guangxi University, Nanning, Guangxi, China

**Keywords:** *Glenea cantor*, longicorn beetle, Lamiinae, mitochondrial genome

## Abstract

The complete mitogenome sequence of an Asian longicorn beetle *Glenea cantor*, was sequenced. The 15,514 bp long genome has the standard metazoan complement of 38 genes. These genes contain 13 protein-coding genes, 22 transfer RNA genes, 2 ribosomal RNA genes, and 1 control region. The nucleotide composition of the *G. cantor* mitogenome was A: 39.4%, T: 37.7%, G: 8.9%, C: 14.0%. The A + T content is 77.1%, showing strong AT skew. Phylogenetic analysis indicated that *G. cantor* have sister relationship with *Thyestilla gebleri*.

## Introduction

Asian longicorn beetle *Glenea cantor* (Coleoptera: Cerambycidae: Lamiinae) is a vital pest on the kapok trees (*Gossampinus malabaricus* DC.) in South East Asian countries (Lu et al. 2011a). The larvae grow inside the trunks or branches by chewing cellulose, which kill branches and finally whole trees (Lai et al. 2008). Moreover, it has high reproductive ability (Lu et al. 2013) and its host range makes it a possible invasive pest outside Asia if established (Lu, et al. 2011b). Mitochondrial genome sequences are essential to a deeper understanding of the evolution of Cerambycidae and identify larva species (Liu et al. 2018; Wang and Tang 2018). Here, the complete mitochondrial DNA (mtDNA) genome of *G. cantor* was elucidated which has not been reported before.

In this study, specimens of *G. cantor* were collected from the Qingxiu Mountain (22°47′N, 108°23′E) of Nanning City (Guangxi Province, China). The total genomic DNA was extracted following the modified CTAB DNA extraction protocol and stored at Guangxi Key Laboratory of Agric-Environment and Agric-Products Safety (The city of Nanning, China) with sample number of SZHT0410G043. Then library was constructed and pair-end was sequenced (2*150 bp) with HiSeq (Illumina, San Diego, CA). Approximately 11.32 G of raw data and 11.29 G of clean data were obtained for sequence assembly by SPAdes (version 3.9) (Bankevich et al. 2012).

The complete mitochondrial genome of *G. cantor* is a closed circular molecule 15,514 bp in length (GenBank accession number MN044086) and constitutive of 38 genes. These genes contain 13 protein-coding genes (PCGs), 22 transfer RNA (tRNA) genes, 2 ribosomal RNA (rRNA) genes, and 1 control region (D-loop). The single non-coding control region (D-Loop) is 863 bp in length. The nucleotide composition of the *G. cantor* mitogenome was A (39.4%), T (37.7%), G (8.9%), C (14.0%). The A + T content is 77.1%, showing strong AT skew.

**Figure 1.**
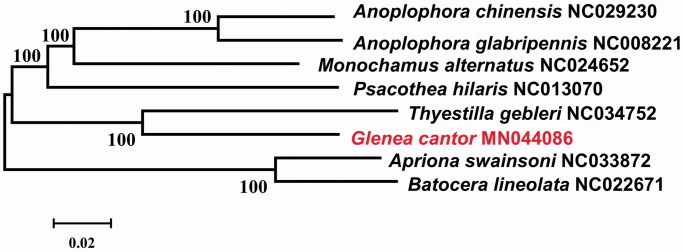
Neighbor-joining phylogenetic tree of *Glenea cantor* and other Lamiinae beetles. The complete mitochondrial genome was downloaded from GenBank and the phylogenic tree was constructed by Neighbor-Joining method with 1000 bootstrap replicates. MtDNA accession numbers used for tree construction are as follows: *Glenea cantor* (MN044086) *Anoplophora chinensis* (NC029230), *Anoplophora glabripennis* (NC008221), *Apriona swainsoni* (NC033872), *Batocera lineolata* (NC022671), *Monochamus alternatus* (NC024652), *Psacothea hilaris* (NC013070), *Thyestilla gebleri* (NC034752).

Molecular Evolutionary Genetics Analysis Version 6.0 (MEGA6.0) was used to make phylogenetic analysis among Lamiinae species by Neighbor-Joining method with 1000 bootstrap replicates (Tamura et al. 2013). The results showed that mtDNA of *G. cantor* had a close relationship with that of *Thyestilla gebleri* (Figure 1).
